# Association between labor duration in transvaginal deliveries and the risk of neonatal intensive care unit admission for newborns

**DOI:** 10.3389/fped.2025.1590830

**Published:** 2025-06-10

**Authors:** Huajuan Chen, Chunyi Zhou, Xiaomeng Yan, Hui Shao

**Affiliations:** ^1^ Department of Delivery Room, Shaoxing Maternity and Child Health Care Hospital, Shaoxing, China; ^2^ Department of Gynecology and Obstetrics, Shaoxing Maternity and Child Health Care Hospital, Shaoxing, China; ^3^ Department of Infectology, Shaoxing Maternity and Child Health Care Hospital, Shaoxing, China

**Keywords:** labor stage first, labor stage second, newborns, NICU, PSM, RCS

## Abstract

**Objective:**

To investigate the relationship between the duration of the first and second stages of labor and the risk of neonatal admission to the neonatal intensive care unit (NICU) among women undergoing vaginal delivery, with the aim of optimizing labor duration to mitigate adverse neonatal outcomes.

**Methods:**

A retrospective study was conducted to analyze baseline data from 13,480 singleton mothers and newborns who underwent vaginal delivery at a tertiary maternity hospital in Zhejiang Province between January 2021 and December 2023. Propensity score matching (PSM) was utilized to adjust for 12 confounding factors that could influence adverse neonatal outcomes, excluding the durations of the first and second stages of labor. Both stages of labor were categorized into quartiles. Single-factor and multifactor logistic regression analyses were performed before and after PSM to investigate the relationship between labor duration and the risk of neonatal NICU admission. Additionally, multi-model logistic regression analyses further examined this relationship. Restricted cubic spline (RCS) plots were generated before and after PSM to assess any non-linear associations between the durations of the first and second stages of labor and NICU admission risk. Subgroup analyses were also conducted to explore how labor duration impacts NICU admission risk across different population segments.

**Results:**

Out of 13,480 neonates, 763 were admitted to the NICU. Multi-model logistic regression analyses indicated that longer durations of the second stage of labor, both before and after matching, were positively associated with an increased risk of NICU admission. In contrast, longer first stage labor durations did not correlate with higher admission risk. Additionally, the RCS analysis revealed a nonlinear relationship between the duration of the second stage of labor and the risk of neonatal NICU admission. Subgroup analyses confirmed that extended second stage labor duration was linked to the risk of NICU admission across various population segments.

**Conclusion:**

Within a certain range, a longer duration of the second stage of labor was associated with an increased risk of neonatal NICU admission. However, no significant correlation was found between the duration of the first stage of labor and the risk of neonatal NICU admission.

## Introduction

Optimizing time-to-delivery management in obstetrics is a continual challenge focused on reducing emergency caesarean section rates and preventing adverse maternal and neonatal outcomes (1). Conversely, NICU admissions, a critical aspect of negative neonatal outcomes, can result in illness and mortality, imposing significant emotional and financial strains on families, as well as substantial costs on the healthcare system (2).

The impact of labor duration on neonatal outcomes has become a significant area of research in recent years. Labor is divided into three stages: the first stage encompasses the period from the onset of regular contractions to full cervical dilation; the second stage, known as the fetal delivery stage, spans from complete dilation to the birth of the fetus (3); and the third stage involves the delivery of the placenta, occurring after the fetus is born. Most research has concentrated on the duration of the second stage of labor. While numerous studies have examined its influence on neonatal outcomes, consensus remains elusive (4). Some research indicates that a prolonged second stage increases the risk of adverse neonatal outcomes (5, 6), while others find no correlation (7, 8). Additionally, there is a notable lack of studies in China and Asia addressing the effects of labor duration on neonatal outcomes, with the impact of the first stage of labor often receiving insufficient attention from researchers.

With this in mind, we conducted a large cross-sectional study to simultaneously investigate the effects of the first and second stages of labor on adverse neonatal outcomes at the same baseline level. Neonatal outcomes were defined by NICU admissions, which indicate adverse results. Unlike previous studies, we aimed for a clearer examination of labor duration's impact on neonates by controlling for multiple confounders through PSM and employing RCS to analyze the non-linear relationship between labor duration and adverse outcomes. Our results revealed a non-linear correlation between the duration of the second stage of labor and adverse neonatal outcomes.

## Methods

### The study population

**Figure 1:**
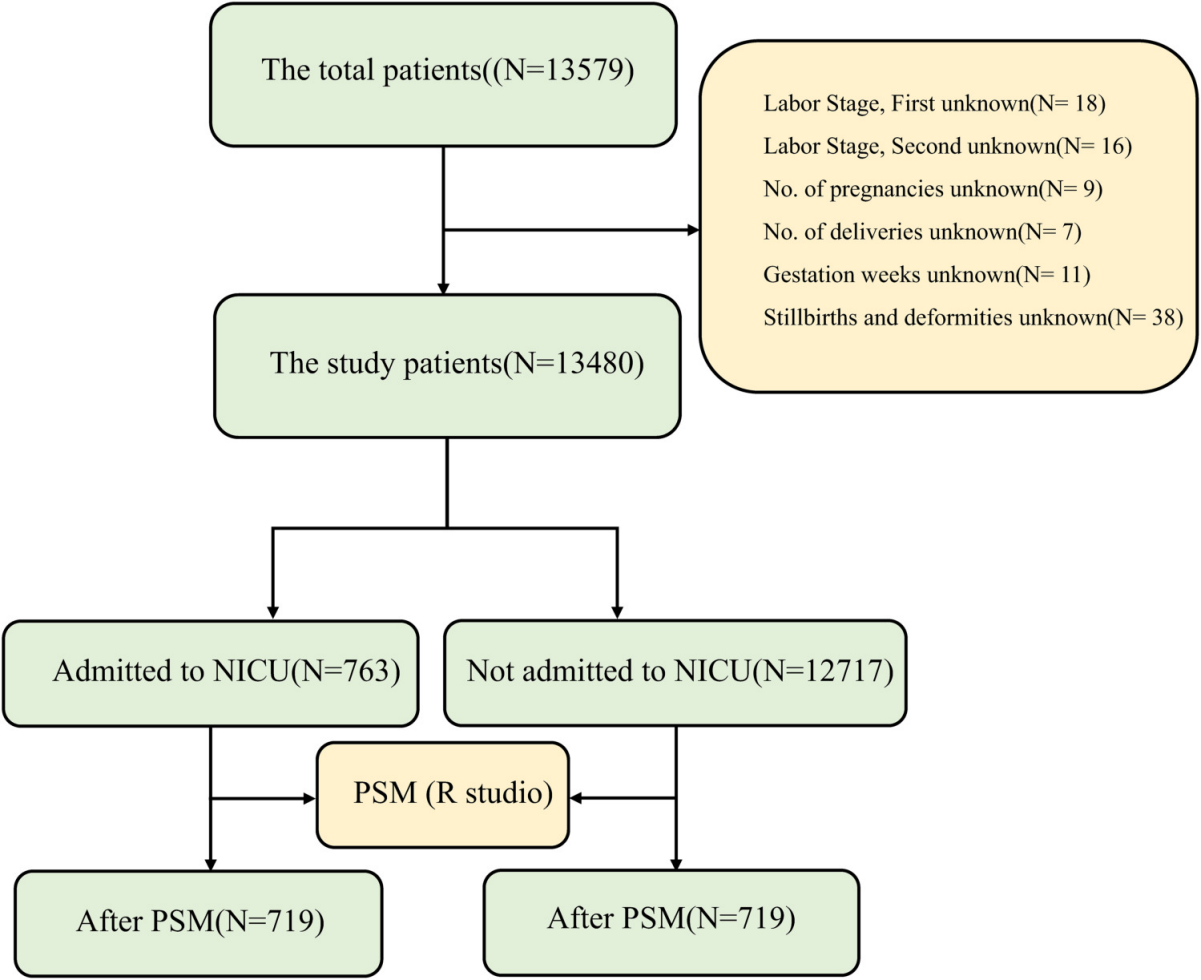
Flowchart illustrating the participant screening process for study inclusion. NICU, neonatal intensive care unit; PSM, propensity score matching.

The study population comprised 13,480 singleton mothers admitted to our hospital between January 2021 and December 2023, along with 13,480 newborns delivered via natural or vaginally assisted methods. Figure 1 illustrates the flowchart for population screening and inclusion in the study.

### Data collection

We conducted a retrospective analysis of basic maternal and neonatal characteristics using data from the existing electronic medical record and nursing documentation systems. The factors collected included maternal age, gestational age, number of births, mode of delivery, and the presence of high-risk factors, which encompassed conditions such as pregnancy-related diseases, advanced maternal age, pre-eclampsia, low amniotic fluid, preterm premature rupture of membranes, and obstetric anomalies. The identification of high-risk factors was determined by experienced obstetricians, midwives, and neonatologists based on several criteria: blood loss during labor, duration of the first and second stages of labor, newborn sex, birth weight, and NICU admission status. Due to significant missing data, maternal BMI was excluded from the analysis. Additionally, the third stage of labor was not analyzed, as the delivery occurred during this stage and it did not influence adverse neonatal outcomes.

### Statistical analysis

Descriptive analyses of participant characteristics were conducted, and all data were statistically analyzed using R software version 4.4.1. Continuous variables were reported as means and standard deviations (SD), assessed with t-tests for baseline characteristics, while categorical variables were expressed as percentages and analyzed using chi-square tests. We employed the PSM method with a 1:1 ratio to balance the case and control groups, a common approach to enhance statistical power and minimize bias in observational studies (9–11). Multi-model logistic regression analysis evaluated the relationship between the duration of the first and second stages of labor and neonatal admission to the NICU, with these durations treated as continuous variables divided into three levels based on quartile cut-offs, using the first level as the reference group. RCS analysis was performed to explore the non-linear relationship between labor duration and NICU admission risk. Subgroup analyses considered maternal age (pre- and post-PSM), number of pregnancies, number of births, week of gestation, mode of delivery, hemorrhage amount, labor assistance, oxytocin use, artificial rupture of membranes, presence of high-risk factors, and infant sex and weight. Statistical significance was defined as *p* < 0.05.

## Results

### Baseline characteristics of confounding variables before and after PSM in the study population

**Figure 2:**
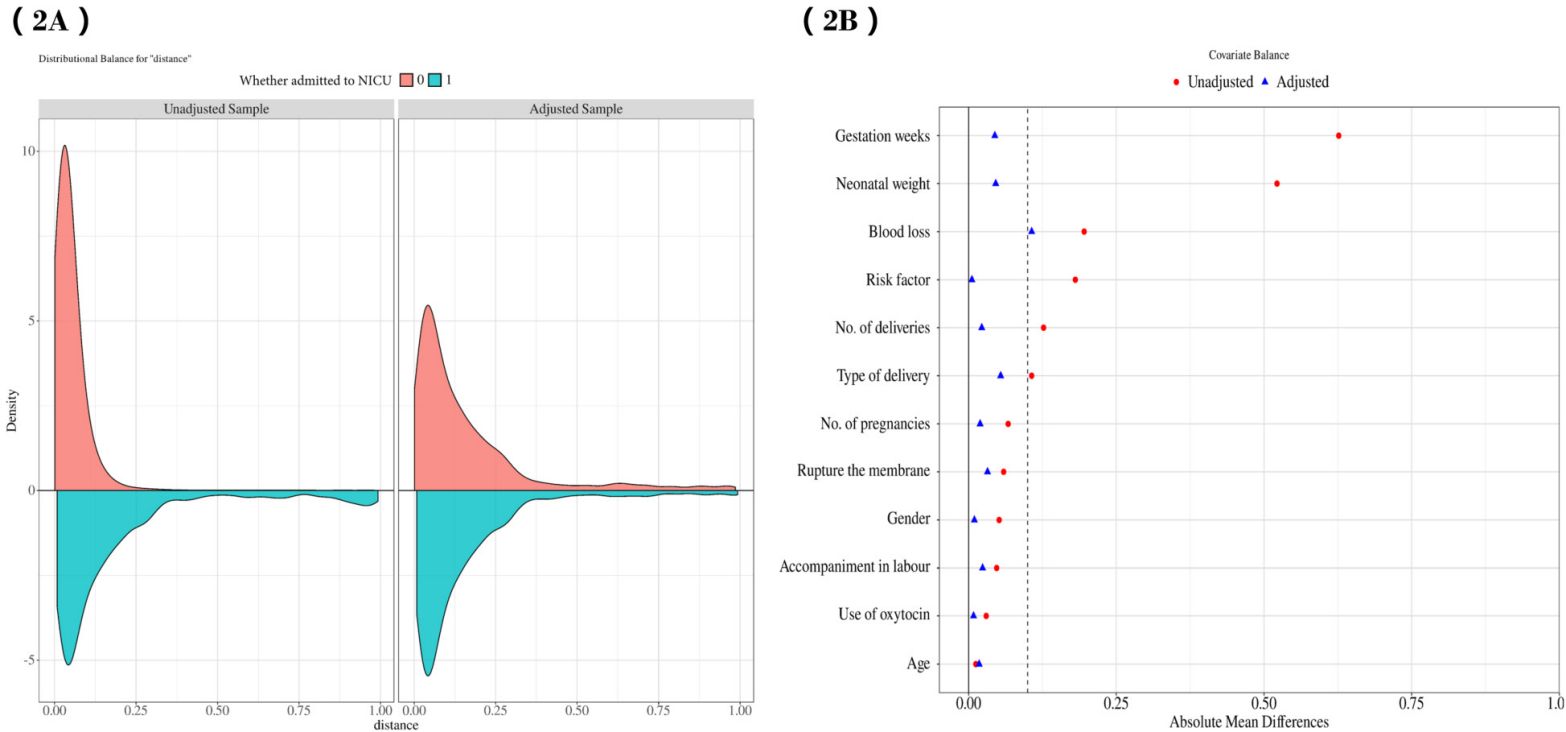
**(A)** illustrates the data distribution both before and after PSM, while **(B)** presents the SMD values prior to and following the matching process. PSM, propensity score matching; SMD, standardized mean differences.

A total of 13,480 participants were included in the study, and their data were statistically analyzed. Table 1 presents the baseline characteristics of maternal and neonatal confounders based on NICU admission status. Mothers of newborns admitted to the NICU had shorter gestational weeks, were more likely to be primigravida, had fewer pregnancies, required assisted vaginal delivery, lacked labor support, and presented with higher risk factors compared to mothers of healthy infants. Additionally, lower-weight male infants were more frequently admitted to the NICU. To further examine the relationship between labor duration and neonatal NICU admission, Table 1 also details the characteristics of 12 confounders at baseline after nearest neighbor PSM (1:1), revealing no significant differences between the two groups in most characteristics post-PSM (*p* > 0.05). Figures 2A,B illustrate the data distribution and standardized mean differences (SMD) values before and after matching.

**Table 1:** Baseline characteristics of the study population before and after PSM.

Variable	Before PSM				After PSM			
	Total (*n* = 13,480)	Not to NICU (*n* = 12,717)	To NICU (*n* = 763)	*P*	Total (*n* = 1,438)	Not to NICU (*n* = 719)	To NICU (*n* = 719)	*P*
Age, years (mean, ±SD)	28.76 ± 4.03	28.76 ± 4.01	28.71 ± 4.29	0.720	28.68 ± 4.12	28.71 ± 4.03	28.64 ± 4.21	0.725
Gestation weeks (mean, ±SD)	39.18 ± 1.57	39.31 ± 1.26	37.07 ± 3.56	**<**.**001**	37.64 ± 2.89	37.72 ± 2.79	37.56 ± 2.98	0.301
Neonatal weight, kg (mean, ±SD)	3.28 ± 0.44	3.31 ± 0.40	2.89 ± 0.79	**<**.**001**	3.00 ± 0.68	3.02 ± 0.65	2.98 ± 0.70	0.313
Blood loss, ml (mean, ±SD)	221.45 ± 125.37	219.97 ± 124.68	246.19 ± 134.06	**<**.**001**	253.58 ± 163.35	260.74 ± 188.93	246.43 ± 132.70	0.097
No. of pregnancies, *n* (%)				**<**.**001**				0.459
<2	6,296 (46.71)	5,892 (46.33)	404 (52.95)		780 (54.24)	397 (55.22)	383 (53.27)	
≥2	7,184 (53.29)	6,825 (53.67)	359 (47.05)		658 (45.76)	322 (44.78)	336 (46.73)	
No. of deliveries, *n* (%)				**<**.**001**				0.332
Primipara	8,366 (62.06)	7,801 (61.34)	565 (74.05)		1,074 (74.69)	545 (75.80)	529 (73.57)	
Multipara	5,114 (37.94)	4,916 (38.66)	198 (25.95)		364 (25.31)	174 (24.20)	190 (26.43)	
Type of delivery, *n* (%)				**<**.**001**				**0**.**014**
Natural birth	12,201 (90.51)	11,587 (91.11)	614 (80.47)		1,113 (77.4)	537 (74.69)	576 (80.11)	
Non- natural birth	1,279 (9.49)	1,130 (8.89)	149 (19.53)		325 (22.6)	182 (25.31)	143 (19.89)	
Gender, *n* (%)				**0**.**005**				0.710
Male infant	6,915 (51.3)	6,486 (51.00)	429 (56.23)		797 (55.42)	395 (54.94)	402 (55.91)	
Female infant	6,565 (48.7)	6,231 (49.00)	334 (43.77)		641 (44.58)	324 (45.06)	317 (44.09)	
Accompaniment in labour,n (%)				**<**.**001**				0.239
No	2,210 (16.39)	2,051 (16.13)	159 (20.84)		253 (17.59)	118 (16.41)	135 (18.78)	
Yes	11,270 (83.61)	10,666 (83.87)	604 (79.16)		1,185 (82.41)	601 (83.59)	584 (81.22)	
Rupture the membrane, *n* (%)				**0**.**001**				0.225
Naturally	6,165 (45.73)	5,773 (45.40)	392 (51.38)		755 (52.5)	389 (54.10)	366 (50.90)	
Man-made	7,315 (54.27)	6,944 (54.60)	371 (48.62)		683 (47.5)	330 (45.90)	353 (49.10)	
Use of oxytocin, *n* (%)				0.117				0.746
No	6,094 (45.21)	5,770 (45.37)	324 (42.46)		572 (39.78)	283 (39.36)	289 (40.19)	
Yes	7,386 (54.79)	6,947 (54.63)	439 (57.54)		866 (60.22)	436 (60.64)	430 (59.81)	
Risk factor, *n* (%)				**<**.**001**				0.737
No	9,804 (72.73)	9,119 (71.71)	685 (89.78)		1,278 (88.87)	637 (88.60)	641 (89.15)	
Yes	3,676 (27.27)	3,598 (28.29)	78 (10.22)		160 (11.13)	82 (11.40)	78 (10.85)	

NICU, neonatal intensive care unit; PSM, propensity score matching; SD, standard deviations.

Bold values indicate *p* < 0.05 signifying statistical significance.

### Association between the duration of the first and second stage of labour and the risk of neonatal admission to the NICU

#### Univariate and multivariate logistic regression analyses of the association between labor duration and the risk of neonatal admission to the NICU

Table 2 presents the results of univariate and multivariate logistic regression analyses regarding the likelihood of newborns being admitted to the NICU. Univariate logistic regression was employed to explore the relationships between various factors—such as maternal age, gestational age, number of births, delivery mode, amount of hemorrhage, whether labor was assisted, oxytocin use, artificial rupture of membranes, presence of high-risk factors, and the baby's sex and weight—and the risk of NICU admission. Our findings indicated that gestational week was negatively associated with the risk of NICU admission [0.62 (0.60–0.65)], while fetal weight also showed a negative correlation [0.20 (0.18–0.23)]. Conversely, maternal hemorrhage was positively associated with NICU admission risk [1.01 (1.01–1.01)]. Interestingly, while a higher number of pregnancies generally decreased the risk of postnatal NICU admission [0.77 (0.66–0.89)], it was found that neonates born via assisted vaginal delivery had a higher risk of NICU admission [2.49 (2.06–3.01)], whereas female infants had a lower risk [0.81 (0.70–0.94)]. Moreover, mothers who were accompanied during labor had a reduced risk of their newborns being admitted to the NICU [0.73 (0.61–0.88)], and those who underwent artificial rupture of membranes also experienced a lower risk [0.79 (0.68–0.91)]. In contrast, mothers with high-risk factors had an increased likelihood of their infants being admitted to the NICU [3.47 (2.73–4.39)].The risk of NICU admission was significantly higher for cases where both the first and second stages of labor were at the Q4 level [OR: 1.40; 95% CI: (1.14–1.71) and OR: 1.52; 95% CI: (1.23–1.88), respectively] (*p* < 0.05). Multivariate logistic regression further revealed that the risk of NICU admission remained elevated for fetuses during both labor stages at the Q4 level [OR: 1.46; 95% CI: (1.13–1.90) and [OR: 2.10; 95% CI: (1.51–2.92)] (*p* < 0.05). After PSM, both univariate and multivariate analyses indicated a heightened risk of NICU admission during the second stage of labor at the Q4 level [OR: 1.41; 95% CI: (1.04–1.90) and OR: 2.46; 95% CI: (1.62–3.73)] (*p* < 0.05). Notably, no significant difference was observed at the Q4 level for the first stage of labor.

**Table 2:** Univariate and multivariate logistic regression analysis of the association between labor duration and neonatal admission risk to the NICU.

Variable	Before PSM				After PSM			
	Univariate logistic		Multivariable logistic		Univariate logistic		Multivariable logistic	
	OR (95% CI)	*P*	OR (95% CI)	*P*	OR (95% CI)	*P*	OR (95% CI)	*P*
Age, years (mean, ±SD)	1.00 (0.98∼1.01)	0.704	1.00 (0.98∼1.02)	0.852	1.00 (0.97∼1.02)	0.725	0.99 (0.96∼1.01)	0.312
Gestation weeks (mean, ±SD)	0.62 (0.60∼0.65)	**<**.**001**	0.60 (0.56∼0.64)	**<**.**001**	0.98 (0.95∼1.02)	0.301	0.98 (0.91∼1.05)	0.537
Neonatal weight, kg (mean, ±SD)	0.20 (0.18∼0.23)	**<**.**001**	0.94 (0.74∼1.20)	0.637	0.92 (0.79∼1.08)	0.313	0.93 (0.70∼1.25)	0.639
Blood loss, ml (mean, ±SD)	1.01 (1.01∼1.01)	**<**.**001**	1.01 (1.01∼1.01)	**<**.**001**	1.00 (1.00∼1.00)	0.101	1.00 (1.00∼1.00)	0.138
No. of pregnancies, *n* (%)								
<2	1.00 (Reference)		1.00 (Reference)		1.00 (Reference)		1.00 (Reference)	
≥2	0.77 (0.66∼0.89)	**<**.**001**	1.15 (0.93∼1.42)	0.193	1.08 (0.88∼1.33)	0.459	1.03 (0.78∼1.36)	0.818
No. of deliveries, *n* (%)								
Primipara	1.00 (Reference)		1.00 (Reference)		1.00 (Reference)		1.00 (Reference)	
Multipara	0.56 (0.47∼0.66)	**<**.**001**	0.83 (0.62∼1.12)	0.215	1.12 (0.89∼1.43)	0.332	1.52 (1.03∼2.25)	0.035
Type of delivery, *n* (%)								
Natural birth	1.00 (Reference)		1.00 (Reference)		1.00 (Reference)		1.00 (Reference)	
Non- natural birth	2.49 (2.06∼3.01)	**<**.**001**	2.21 (1.78∼2.75)	**<**.**001**	0.73 (0.57∼0.94)	**0**.**014**	0.67 (0.50∼0.88)	**0**.**004**
Gender, *n* (%)								
Male infant	1.00 (Reference)		1.00 (Reference)		1.00 (Reference)		1.00 (Reference)	
Female infant	0.81 (0.70∼0.94)	**0**.**005**	0.92 (0.78∼1.09)	0.335	0.96 (0.78∼1.18)	0.710	0.99 (0.80∼1.22)	0.912
Accompaniment in labour, *n* (%)								
No	1.00 (Reference)		1.00 (Reference)		1.00 (Reference)		1.00 (Reference)	
Yes	0.73 (0.61∼0.88)	**<**.**001**	0.84 (0.66∼1.08)	0.169	0.85 (0.65∼1.11)	0.239	0.85 (0.62∼1.16)	0.310
Rupture the membrane, *n* (%)								
Naturally	1.00 (Reference)		1.00 (Reference)		1.00 (Reference)		1.00 (Reference)	
Man-made	0.79 (0.68∼0.91)	**0**.**001**	1.18 (0.99∼1.39)	0.059	1.14 (0.92∼1.40)	0.225	1.16 (0.93∼1.45)	0.188
Use of oxytocin, *n* (%)								
No	1.00 (Reference)		1.00 (Reference)		1.00 (Reference)		1.00 (Reference)	
Yes	1.13 (0.97∼1.30)	0.117	1.20 (1.00∼1.45)	**0**.**052**	0.97 (0.78∼1.19)	0.746	0.93 (0.73∼1.19)	0.586
Risk factor, *n* (%)								
No	1.00 (Reference)		1.00 (Reference)		1.00 (Reference)		1.00 (Reference)	
Yes	3.47 (2.73∼4.39)	**<**.**001**	1.91 (1.48∼2.46)	**<**.**001**	1.06 (0.76∼1.47)	0.737	1.21 (0.85∼1.73)	0.282
Labor stage, first, *n* (%)								
Q1	1.00 (Reference)		1.00 (Reference)		1.00 (Reference)		1.00 (Reference)	
Q2	0.80 (0.64∼1.01)	0.056	0.90 (0.69∼1.17)	0.436	0.90 (0.65∼1.24)	0.510	0.95 (0.67∼1.33)	0.758
Q3	1.01 (0.82∼1.25)	0.910	1.12 (0.86∼1.45)	0.394	0.89 (0.66∼1.20)	0.430	0.92 (0.66∼1.29)	0.646
Q4	1.40 (1.14∼1.71)	**0**.**001**	1.46 (1.13∼1.90)	**0**.**004**	1.22 (0.91∼1.63)	0.185	1.33 (0.94∼1.88)	0.112
Labor stage, second, *n* (%)								
Q1	1.00 (Reference)		1.00 (Reference)		1.00 (Reference)		1.00 (Reference)	
Q2	1.21 (0.97∼1.51)	0.083	1.62 (1.22∼2.15)	**<**.**001**	1.31 (0.95∼1.79)	0.095	1.71 (1.21∼2.43)	**0**.**002**
Q3	1.16 (0.93∼1.45)	0.182	1.68 (1.22∼2.30)	**0**.**001**	1.12 (0.82∼1.54)	0.462	1.71 (1.15∼2.55)	**0**.**008**
Q4	1.52 (1.23∼1.88)	**<**.**001**	2.10 (1.51∼2.92)	**<**.**001**	1.41 (1.04∼1.90)	**0**.**026**	2.46 (1.62∼3.73)	**<**.**001**

CI, confidence intervals; OR, odds ratios; Quartiles: Q1 (0%–25%), Q2 (25%–50%), Q3 (50%–75%), Q4 (75%–100%); NICU, neonatal intensive care unit; PSM, propensity score matching.

Bold values indicate *p* < 0.05 signifying statistical significance.

#### Multimodal logistic regression analysis of the relationship between labor duration and the risk of neonatal admission to the NICU

Three logistic regression models were developed to examine the relationship between the duration of the first and second stages of labor and the risk of neonatal admission to the NICU, as detailed in Table 3. Model 1 was a crude model without covariate adjustments. Model 2 adjusted for age, gestational week, number of pregnancies and deliveries. Model 3 was a fully adjusted model that includes and accounts for all relevant covariates. Results indicated a positive association between neonatal NICU admission risk and both stages of labor across all models, with odds ratios (OR) and 95% confidence intervals (CI) of 1.05 (1.03–1.08), 1.07 (1.05–1.10), and 1.06 (1.03–1.09) for the first stage (*p* < 0.05). For the second stage, the OR and CI were 1.13 (1.06–1.20), 1.18 (1.10–1.27), and 1.09 (1.01–1.18) (*p* < 0.05). At the quartile 4 (Q4) level, the first stage showed significant differences with OR of 1.40 (1.14–1.71), 1.64 (1.28–2.10), and 1.46 (1.13–1.90) (*p* < 0.005). Similarly, the second stage displayed significant differences at Q4 with OR of 1.52 (1.23–1.88), 2.59 (1.88–3.55), and 2.10 (1.51–2.92) (*p* < 0.05). These findings suggest that prolonged duration of the second stage of labor may serve as an independent risk factor for neonatal NICU admission. Even after adjustments in the three models, a significant association between the second stage at Q4 level and increased neonatal admission risk persisted, with OR of 1.41 (1.04–1.90), 2.06 (1.38–3.08), and 2.46 (1.62–3.73) (*p* < 0.05), highlighting a strengthened relationship.

**Table 3:** Multi-model logistic regression analysis of the relationship between labor duration and the risk of neonatal admission to the NICU.

Model	Variable	Characteristic	Before PSM		After PSM	
			OR (95% CI)	*P*	OR (95% CI)	*P*
Model 1	Labor stage, first	Total	1.05 (1.03∼1.08)	**<**.**001**	1.02 (0.99∼1.05)	0.132
		Q1	1.00 (Reference)		1.00 (Reference)	
		Q2	0.80 (0.64∼1.01)	0.056	0.90 (0.65∼1.24)	0.510
		Q3	1.01 (0.82∼1.25)	0.910	0.89 (0.66∼1.20)	0.430
		Q4	1.40 (1.14∼1.71)	**0**.**001**	1.22 (0.91∼1.63)	0.185
	Labor stage, second	Total	1.13 (1.06∼1.20)	**<**.**001**	1.07 (0.95∼1.20)	0.251
		Q1	1.00 (Reference)		1.00 (Reference)	
		Q2	1.21 (0.97∼1.51)	0.083	1.31 (0.95∼1.79)	0.095
		Q3	1.16 (0.93∼1.45)	0.182	1.12 (0.82∼1.54)	0.462
		Q4	1.52 (1.23∼1.88)	**<**.**001**	1.41 (1.04∼1.90)	**0**.**026**
Model 2	Labor stage, first	Total	1.07 (1.05∼1.10)	**<**.**001**	1.03 (1.01∼1.07)	**0**.**033**
		Q1	1.00 (Reference)		1.00 (Reference)	
		Q2	0.91 (0.70∼1.17)	0.453	0.90 (0.64∼1.25)	0.526
		Q3	1.16 (0.90∼1.49)	0.260	0.88 (0.64∼1.22)	0.448
		Q4	1.64 (1.28∼2.10)	**<**.**001**	1.24 (0.89∼1.72)	0.204
	Labor stage, second	Total	1.18 (1.10∼1.27)	**<**.**001**	1.16 (1.01∼1.33)	**0**.**031**
		Q1	1.00 (Reference)		1.00 (Reference)	
		Q2	1.65 (1.25∼2.18)	**<**.**001**	1.65 (1.17∼2.33)	**0**.**005**
		Q3	1.73 (1.26∼2.36)	**<**.**001**	1.61 (1.09∼2.38)	**0**.**017**
		Q4	2.59 (1.88∼3.55)	**<**.**001**	2.06 (1.38∼3.08)	**<**.**001**
Model 3	Labor stage, first	Total	1.06 (1.03∼1.09)	**<**.**001**	1.04 (1.01∼1.07)	**0**.**024**
		Q1	1.00 (Reference)		1.00 (Reference)	
		Q2	0.90 (0.69∼1.17)	0.436	0.95 (0.67∼1.33)	0.758
		Q3	1.12 (0.86∼1.45)	0.394	0.92 (0.66∼1.29)	0.646
		Q4	1.46 (1.13∼1.90)	**0**.**004**	1.33 (0.94∼1.88)	0.112
	Labor stage, second	Total	1.09 (1.01∼1.18)	**0**.**030**	1.26 (1.09∼1.46)	**0**.**002**
		Q1	1.00 (Reference)		1.00 (Reference)	
		Q2	1.62 (1.22∼2.15)	**<**.**001**	1.71 (1.21∼2.43)	**0**.**002**
		Q3	1.68 (1.22∼2.30)	**0**.**001**	1.71 (1.15∼2.55)	**0**.**008**
		Q4	2.10 (1.51∼2.92)	**<**.**001**	2.46 (1.62∼3.73)	**<**.**001**

CI, confidence intervals; OR, odds ratios; Quartiles: Q1 (0%–25%), Q2 (25%–50%), Q3 (50%–75%), Q4 (75%–100%); NICU, neonatal intensive care unit; PSM, propensity score matching.

Bold values indicate *p* < 0.05 signifying statistical significance.

### Subgroup analyses before and after PSM

**Figure 3:**
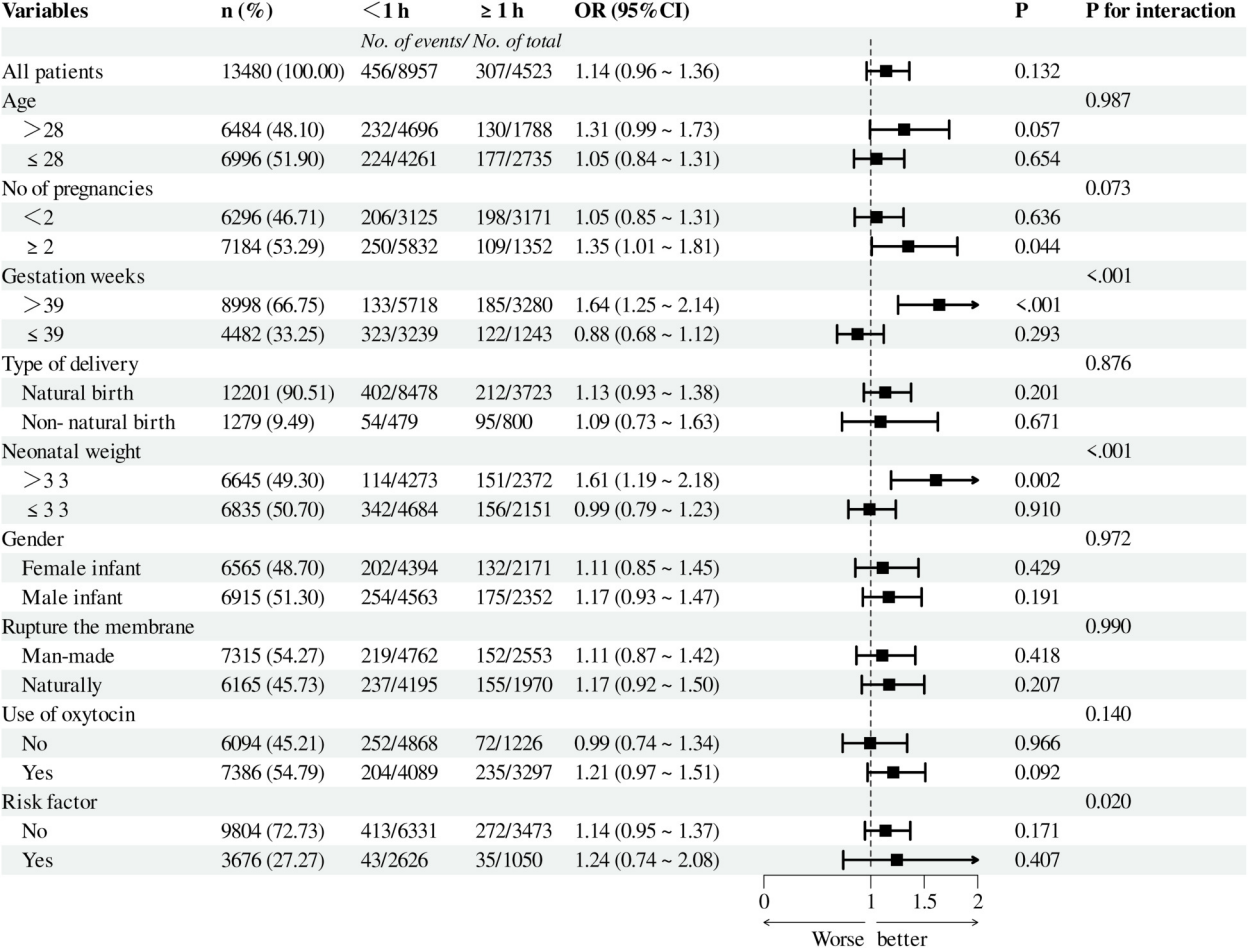
Before PSM, subgroup analysis of the relationship between the duration of the second stage of labor and the risk of NICU admission. A cutoff value of 1 was used to convert the duration of the second stage of labor into a categorical variable. NICU, neonatal intensive care unit; PSM, propensity score matching.

**Figure 4:**
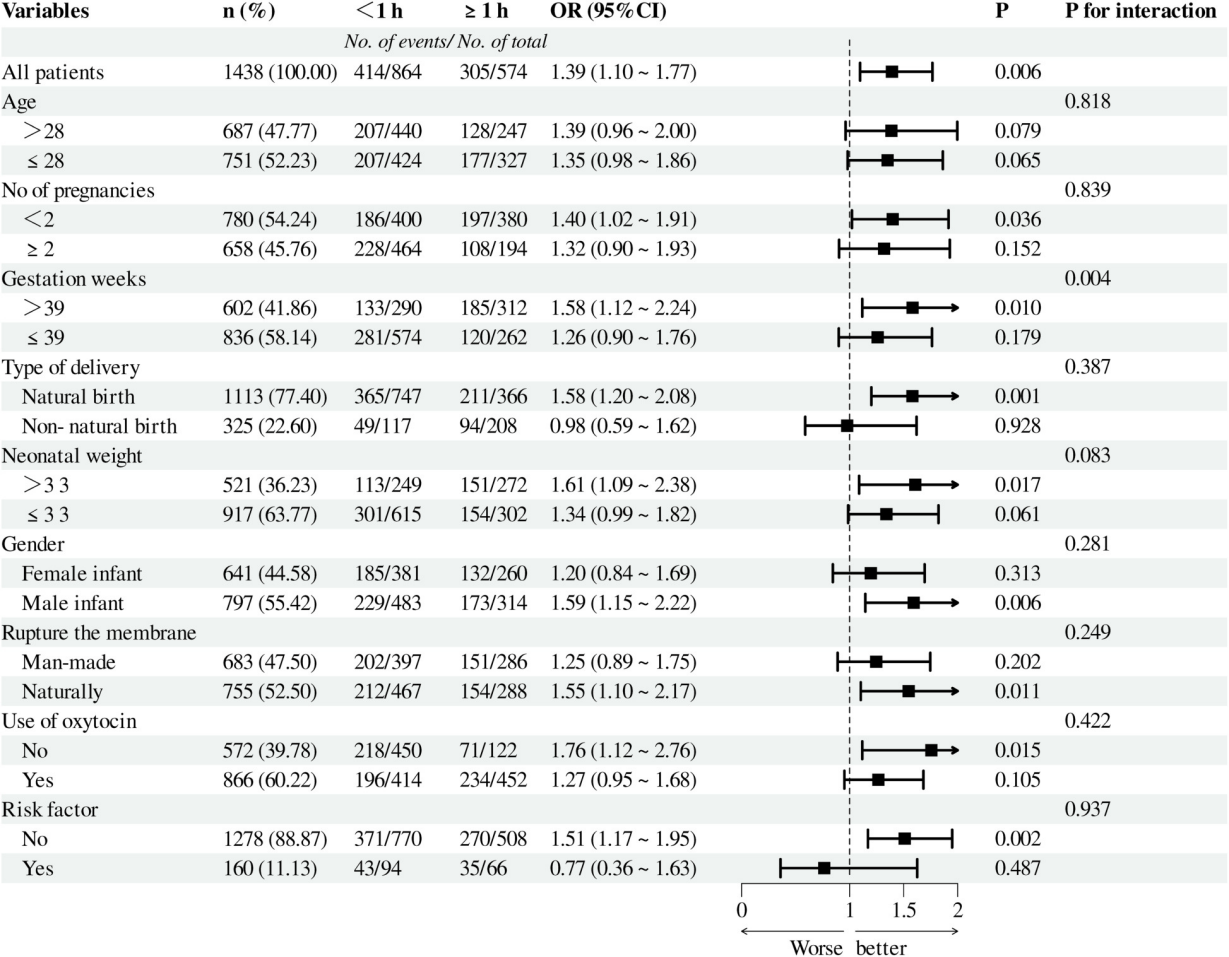
After PSM, subgroup analysis of the relationship between the duration of the second stage of labor and the risk of NICU admission. A cutoff value of 1 was used to convert the duration of the second stage of labor into a categorical variable. NICU, neonatal intensive care unit; PSM, propensity score matching.

To assess the relationship between the duration of the second stage of labor and various factors—such as maternal age, number of pregnancies, week of gestation, mode of delivery, Neonatal weight and gender, use of oxytocin, artificial rupture of membranes, and high-risk factors—we conducted subgroup analyses (Figures 3,4). Pre-PSM, our findings indicated that small for gestational weeks, low birth weight, and high-risk factors could influence the correlation between the duration of the second stage of labor and the risk of neonatal admission to the NICU (interaction *P*-value < 0.05). The results following PSM suggest that only small gestational age may influence this correlation (interaction *P*-value < 0.05). Overall, both pre- and post-PSM analyses revealed that a second stage of labor lasting over one hour is linked to an increased risk of neonatal NICU admission (OR > 1).

### Non-linear relationship between the duration of labor and the risk of neonatal admission to the NICU

**Figure 5:**
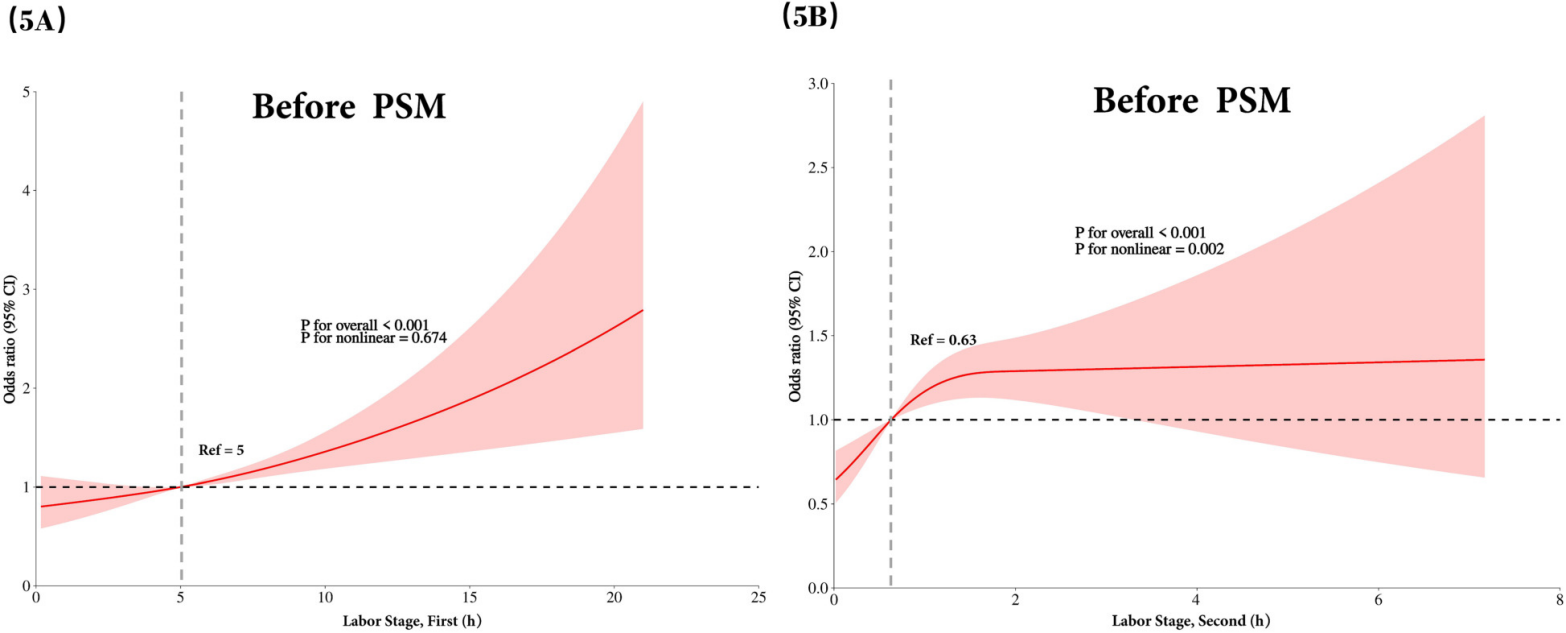
Before PSM, nonlinear correlation plots illustrating the association between labor duration and the risk of neonatal admission to the NICU are presented based on model 3's RCS analysis. **(A)** Depicts the nonlinear correlation between the duration of the first stage of labor and the risk of NICU admission, while **(B)** shows the corresponding plot for the second stage of labor. NICU, neonatal intensive care unit; PSM, propensity score matching; RCS, restricted cubic spline.

**Figure 6:**
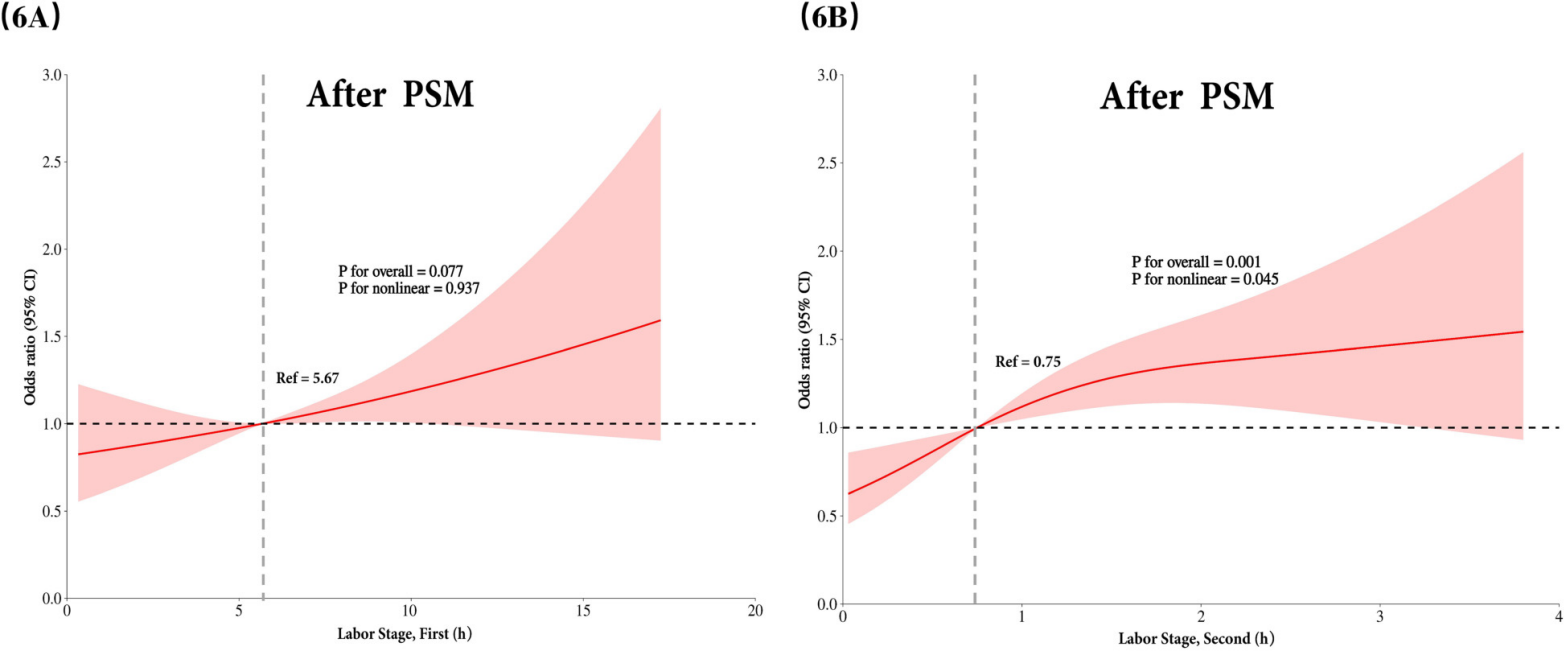
After PSM, nonlinear correlation plots depicting the association between labor duration and the risk of neonatal admission to the NICU are presented based on model 3's RCS analysis. **(A)** Illustrates the nonlinear correlation between the duration of the first stage of labor and the risk of NICU admission, while **(B)** presents the corresponding plot for the second stage of labor. NICU, neonatal intensive care unit; PSM, propensity score matching; RCS, restricted cubic spline.

As illustrated in Figures 5, 6, we utilized Model 3 (the fully corrected model) to plot the RCS and visualize the relationship between the duration of the first and second stages of labor and the risk of neonatal admission to the NICU, both before and after PSM. The findings indicated that there was no nonlinear relationship between the duration of the first stage of labor and the risk of NICU admission at either time point. In contrast, the duration of the second stage of labor demonstrated a nonlinear relationship both before and after PSM. RCS analyses demonstrate that once the second stage exceeds 2 h, the risk does not increase further but remains steady at the initial level.

## Discussion

Using three years of data on spontaneous and vaginally assisted deliveries at our hospital, we explored the relationship between the durations of the first and second stages of labor and the risk of neonatal admission to the NICU. Our analysis revealed a significant difference in labor duration between neonates admitted to the NICU and those who were not. Both pre- and post-PSM univariate and multivariate logistic regression analyses indicated a correlation between longer durations of the second stage of labor and an increased risk of NICU admission, while no significant association was found for the first stage of labor. Furthermore, primiparous women with extended second-stage labor durations were more likely to have their infants admitted to the NICU. Restricted cubic spline analyses confirmed a nonlinear relationship between the duration of the second stage of labor and the risk of neonatal admission, whereas the duration of the first stage did not show a significant association.

The health of newborns is a primary concern in obstetrics and neonatology. Our study indicates that reducing the duration of labor benefits neonates. Previous research (12–14) has established a strong link between prolonged labor and negative maternal outcomes. For instance, Simic et al. found that the risk of severe maternal perineal lacerations increases with longer labor (15). A meta-analysis by Pergialiotis et al. highlighted that an extended second stage of labor correlates with postpartum hemorrhage, chorioamnionitis, endometritis, postpartum fever, and injuries to the obstetric anal sphincter. Additionally, it raises the likelihood of neonatal intensive care unit admission and neonatal sepsis (16). A systematic review (17) showed that a prolonged second stage increases the risk of a 5-minute Apgar score below 7 and NICU admission in first-time mothers, though it does not elevate neonatal death risk. Furthermore, a multicenter study from Germany reported higher neonatal referral rates to the NICU among infants from mothers with prolonged second stages, supporting our findings.

It is crucial to focus on managing labor duration, particularly aiming to minimize the second stage of labor to safeguard the health of both the mother and newborn. Numerous studies have explored methods to shorten labor duration. For instance, Sammour et al. demonstrated that administering intramuscular dexamethasone prior to labor induction significantly reduced the time from the start of induction to the onset of the active phase, as well as the duration of both the active phase and the second stage of labor, without resulting in any maternal or neonatal complications (18). Similarly, Seval MM et al. found that the use of vaginal lubricating gel during labor can reduce labor duration by decreasing friction associated with vaginal delivery, thereby shortening the second stage of labor (19). Additionally, while intravenous glucose infusion during labor may shorten the first stage, it does not appear to affect the second stage (20). A meta-analysis by Schiattarella et al. suggested that smooth muscle antispasmodic agents like resorcinol could effectively reduce the duration of both the first and second stages of labor (21). Furthermore, a prospective study by Luo et al. indicated that epidural analgesia could effectively shorten labor duration while providing pain relief for the mother (22). However, our study did not consider epidural anesthesia as a variable due to insufficient data. Other research has also highlighted that continuous midwifery support for first-time mothers in spontaneous labor can contribute to reduced labor duration (23, 24).

Our study presents several strengths and implications. Notably, unlike previous research, we concurrently examined the effects of the duration of both the first and second stages of labor on the fetus from a consistent baseline. Additionally, we employed PSM to control for confounding variables, thereby enhancing the reliability of our findings. We also conducted stratified subgroup analyses to further explore the association between the duration of the second stage of labor and the risk of neonatal admission to the NICU across different populations, highlighting the need for more tailored preventive strategies. However, our study has certain limitations that must be addressed. Firstly, there is a possibility of inaccuracies in recording the duration of the first stage of labor; for instance, the first stage may have commenced prior to the woman's admission to the emergency department. Moreover, the onset of the second stage was often determined through self-reporting from the woman or her family, which could introduce bias, although such instances are rare. Additionally, the cross-sectional design of our study limits the ability to establish causal relationships. Future prospective studies are necessary to gain a clearer understanding of the connection between the duration of the second stage of labor and the risk of neonatal admission to the NICU, which will be the focus of our upcoming research.

## Conclusion

Our study revealed that an extended duration of the second stage of labor correlates with an increased risk of fetal admission to the NICU. We also examined subgroup differences and identified non-linear relationships, findings that can be highly beneficial in obstetric and neonatal clinical practice.

## Data Availability

The raw data supporting the conclusions of this article will be made available by the authors, without undue reservation.
